# Adherence to the Mediterranean Diet and Metabolic Gene Expression in Smokers: An Integrative Transcriptomic Approach

**DOI:** 10.3390/nu18020276

**Published:** 2026-01-15

**Authors:** İlayda Öztürk Altuncevahir, Ayşe Büşranur Çelik, Kezban Uçar Çifçi, Mervenur Uslu, Meltem Vural, Alev Kural, Ezgi Nurdan Yenilmez Tunoğlu, Yusuf Tutar

**Affiliations:** 1 Department of Nutrition and Dietetics, Faculty of Health Sciences, Bahcesehir University, 34353 Istanbul, Türkiye; ilayda.altuncevahir@bau.edu.tr; 2 Division of Nutrition and Dietetics, Hamidiye Health Sciences Institute, University of Health Sciences, 34147 Istanbul, Türkiye; 3 Division of Medicinal Biochemistry, Department of Basic Medical Sciences, Faculty of Medicine, Recep Tayyip Erdogan University, 53020 Rize, Türkiye; aysebusranur_celik25@erdogan.edu.tr; 4 Department of Molecular Biology and Genetics, Hamidiye Health Sciences Institute, University of Health Sciences, 34668 Istanbul, Türkiye; 5 Division of Basic Sciences and Health, Hemp Research Institute, Yozgat Bozok University, 66900 Yozgat, Türkiye; kezban.u.cifci@bozok.edu.tr; 6 Division of Medicinal Biochemistry, Department of Basic Medical Sciences, University of Health Sciences, 34668 Istanbul, Türkiye; mervenur.al@hotmail.com; 7 Department of Physical Medicine and Rehabilitation, Acibadem Maslak Hospital, 34398 Istanbul, Türkiye; meltem.vural@acibadem.com; 8 Biochemistry Department, University of Health Sciences Bakirkoy Dr. Sadi Konuk Training & Research Hospital, University of Health Sciences, 34147 Istanbul, Türkiye; alev.kural@sbu.edu.tr; 9 Division of Medical Techniques and Services, Vocational School of Health Services, Demiroglu Science University, 34394 Istanbul, Türkiye; ezgi.tunoglu@demiroglu.bilim.edu.tr; 10 Training and Research Hospital, Recep Tayyip Erdogan University, 53020 Rize, Türkiye; 11 Medical Oncology Program, Recep Tayyip Erdogan University, 53100 Rize, Türkiye; 12 Molecular Medicine Program, Recep Tayyip Erdogan University, 53100 Rize, Türkiye

**Keywords:** diet, smoking, metabolism, metabolic pathways, energy metabolism

## Abstract

**Background:** Cigarette smoking disrupts cellular energy metabolism and remains a major global health problem. The Mediterranean diet, characterized by antioxidant and anti-inflammatory properties, has been implicated in the regulation of metabolic pathways. **Objective:** This study aimed to examine the association between adherence to the Mediterranean diet and the expression of energy metabolism-related genes in smokers aged 18–55 years. **Methods:** Smokers were classified according to their Mediterranean Diet Adherence Screener (MEDAS) scores into an adhering group (*n* = 24) and a non-adhering group (*n* = 24). Participant characteristics were recorded, blood samples were collected, and total RNA was isolated. Gene expression analysis was performed using a custom RT-qPCR array targeting energy metabolism-related genes. Pathway enrichment analysis was conducted using EnrichR Reactome 2024, and gene–metabolite relationships were explored using MetaboAnalyst 6.0 to support pathway-level interpretation. **Results:** Smoking was associated with coordinated upregulation of genes involved in glycolysis, glucose transport, lipid metabolism, amino acid metabolism, the pentose phosphate pathway, and redox regulation, consistent with a metabolically stressed state. In contrast, adherence to the Mediterranean diet was associated with lower expression of genes related to glycolytic flux, lipid β-oxidation, and amino acid turnover, alongside relatively higher engagement of tricarboxylic acid cycle-related pathways and reduced activation of redox-associated processes. **Conclusions:** Adherence to the Mediterranean diet was associated with differences in the expression of genes involved in cellular energy metabolism among smokers, suggesting a potential modulatory role of dietary patterns in smoking-related metabolic alterations.

## 1. Introduction

Non-communicable diseases are a growing concern worldwide. The most common causes of these diseases are dietary habits rich in saturated fat and simple sugar, insufficient physical activity, and habits that negatively affect health, such as smoking. All these factors are associated with obesity, hypertension, hyperlipidemia, and insulin resistance. Among the six regions, the World Health Organization (WHO) determined that Europe is the region most affected by non-communicable diseases [1]. In line with Global Burden of Disease (GBD) estimates, non-communicable diseases remain the predominant cause of death in Türkiye [2].

Smoking is considered a significant cause of many diseases and mortality risks. It is predicted that approximately one billion people worldwide are smoking, and one billion more adults will start smoking by 2030 [3]. Furthermore, an average of seven million people die annually due to smoking-related diseases [4]. Tobacco smoking damages almost every organ in the human body. This bad habit causes cancer, cardiovascular diseases, respiratory and other chronic diseases. Cataracts, frequent infections, delayed wound healing, inflammatory bowel diseases, and neurodegenerative diseases such as Alzheimer’s and Parkinson’s are among the health problems with an increasing incidence due to smoking [5,6]. Cigarette smoke contains more than 7000 chemicals. About 69 of these chemicals are considered carcinogens [7]. In addition, it is estimated that there are approximately 1015 oxidative free radicals in the content of one puff of smoke inhaled from cigarettes [8]. There is no specific lower exposure limit for the risk of lung cancer or other tissue tumors due to smoking and/or secondhand smoking. Even brief short-term exposure may lead to the development of these tumors [9]. In addition, recent studies have found that even short-term exposure to secondhand smoke increases the risk of cardiovascular disease [10].

Smoking causes lipid peroxidation via tar molecules in biological membranes. This situation poses a risk for the emergence of cardiovascular diseases (CVDs) [11]. Smoking can cause premature aging by acting through DNA methylation and repair mechanisms and high accumulation of advanced glycation end products (AGEs) due to redox imbalance [12]. In addition, smoking triggers the shortening of telomeres. For example, smoking a pack of cigarettes daily for 40 years accelerates telomere wear, equivalent to aging for 7.4 years [13]. Smokers have 3–4 times more risk of stroke than non-smokers [14]. Similarly, smokers die an average of 13–14 years earlier than non-smokers [15]. In addition, the damage that occurs in children who have been exposed to cigarettes during the fetal period can occur as early as five years of age, with damage to important vessels [16]. The results of the studies associate the occurrence of non-communicable diseases such as Type 2 Diabetes Mellitus (DM), obesity, metabolic syndrome, and non-alcoholic fatty liver disease in many age groups with modifiable risk factors such as smoking and poor dietary habits [17].

Diet, especially the fat content, can affect the body’s inflammatory and immune response [18]. Although nutritional therapy has different types of approaches, diets rich in fruits and vegetables are therapeutic approaches whose effects have been proven by many epidemiological studies [19,20]. The Mediterranean diet is a diet rich in fruits, vegetables, legumes, fish and other seafood, and low-fat dairy products. In particular, it is associated with the control of body weight, the prevention of obesity, and the reduction in cardiovascular disease risk. In addition, the fact that the diet is rich in fruits and vegetables can have a protective effect against the development of different types of cancer by increasing the antioxidant and fiber content [21]. The Mediterranean diet is also rich in phytochemicals, which have antioxidant activity. This antioxidant activity can be protective against cardiovascular diseases by repairing DNA damage and nitric oxide (NO) production by activating protein kinase (AMPK) pathways, activated by phosphatidylinositol-3-Kinase (Pl3K/Akt) and adenosine monophosphate (AMP), improving endothelial function [22]. Fish, one of the primary sources of protein in the Mediterranean diet, is rich in omega-3 fatty acids, eicosapentaenoic acid (EPA), and docosahexaenoic acid (DHA). These bioactive components in fish show many antitumor activities and are used as immunonutrition elements [23]. The primary source of fat in the Mediterranean diet is olive oil. The bioactive components in olive oil are also the subject of studies regarding their effects on human health [24]. The Mediterranean diet pyramid is an officially accepted worldwide dietary guide [25]. Previous studies have shown that cigarette smoking is associated with alterations in the expression of genes involved in key metabolic pathways, including glycolysis, lipid metabolism, mitochondrial respiration, and redox regulation. Smoking-induced upregulation of genes such as *HK2*, *G6PD*, members of the *ACSL* family, and components of the tricarboxylic acid (TCA) cycle has been linked to increased energy demand, oxidative stress, and metabolic reprogramming. These transcriptional alterations may contribute to disrupted metabolic homeostasis and potentially increase cardiometabolic risk in smokers [26,27,28,29]. However, how dietary patterns modulate smoking-associated metabolic gene expression remains insufficiently characterized.

This study was designed to explore whether adherence to the Mediterranean diet is associated with differences in metabolic gene expression within a population exposed to smoking-related metabolic stress. Rather than contrasting smokers and non-smokers, the focus was placed on dietary modulation among smokers, aiming to provide insight into how nutritional patterns may relate to energy metabolism under adverse metabolic conditions.

## 2. Materials and Methods

The authors declare that no artificial intelligence tools or technologies were used in the conception and design of this section.

### 2.1. Selection of Participants

This research was conducted between October and December 2021. An a priori power analysis was performed using GPower software (version 3.1) to determine the required sample size. Based on a two-group comparison (Wilcoxon–Mann–Whitney test), an assumed medium effect size (d = 0.5), a significance level of α = 0.20, and a desired power of 80%, a minimum sample size of 24 participants per group (total *n* = 48) was required. The participants were met at a Training and Research Hospital in Istanbul, Türkiye. The age range of the participants was 18–55. This age range was selected to minimize the confounding effects of aging-related metabolic alterations and menopause. Participants were selected from individuals who do not have any acute or chronic diseases, and do not use any medicine. All participants were selected from smokers. Smokers were defined as individuals who were actively smoking at the time of enrollment and reported daily cigarette consumption. Exercising was defined as participation in moderate-intensity exercise for a minimum of 150 min per week. Menopause was the exclusion criterion for women participants. Participants with morbid obesity were excluded from the study. Body mass index (BMI) was measured in all participants and taken into account as a potential confounder in the interpretation of metabolic gene expression findings.

The participant selection process, eligibility assessment, exclusions, and final group allocation are summarized in a CONSORT-style flow diagram provided as Supplementary Figure S1.

The study participants were divided into two groups: those with dietary habits adhering to the Mediterranean diet (*n*:24) and those not adhering to the Mediterranean diet (*n*:24). Adherence to the Mediterranean diet was defined based on the Mediterranean Diet Adherence Screener (MEDAS), with scores of 7 or higher indicating adherence to the Mediterranean diet and scores below 7 indicating non-adherence. The participants’ general characteristics were obtained with a personal information form. The questions in that form were prepared with the help of similar studies from the literature [30]. The Mediterranean Diet Adherence Screener (MEDAS) was used to determine compliance with the Mediterranean diet [31]. Personal information forms and MEDAS data were obtained through mutual interviews. Peripheral blood samples (10 mL) were collected from each participant under standardized conditions in the biochemistry laboratory of the same hospital. Peripheral blood mononuclear cells have been used as they express 80% of the human-coded genome. Different researchers have indicated that these cells are also suitable for studying the effects of dietary interventions and inflammatory signaling pathways associated with cardiovascular diseases [32,33]. Mediterranean Diet Adherence Scale (MEDAS) is a questionnaire consisting of 14 questions whose validity and reliability study was conducted in Türkiye in 2020 by Pehlivanoglu et al. [34].

### 2.2. Real Time-Quantitative PCR (RT-qPCR) Analysis

To investigate the effects of the Mediterranean diet on energy metabolism in smokers, blood samples were collected from two groups: smokers following a Mediterranean diet (*n* = 24), and smokers not following the Mediterranean diet (*n* = 24). Total RNA was isolated from the blood samples using the innuPREP Blood RNA Isolation Kit (innuSCREEN GmbH, Berlin, Germany), and cDNA synthesis was performed using the Wonder RT-cDNA Kit (Euroclone, Pero, Italy), following the manufacturer’s protocols. Expression levels of genes associated with energy metabolism (listed in Supplementary Table S1) were analyzed using the FluoCycle II SYBR Master Mix (Euroclone, Pero, Italy) on a qTOWER 3 Real-Time PCR Thermal Cyclers (Analytik Jena, Jena, Germany), in accordance with the manufacturer’s instructions. The selected gene panel was designed to encompass key nodes of cellular energy metabolism, including glycolysis, the tricarboxylic acid (TCA) cycle, lipid β-oxidation, amino acid metabolism, and redox balance. These pathways have been consistently associated with smoking-induced metabolic stress and are also known to respond to dietary modulation, particularly in the context of Mediterranean dietary patterns. Gene selection was guided by prior experimental studies and the nutritional genomics literature [15,27]. *GAPDH* and *ACTINB* were used as reference genes, as they have been widely reported to exhibit stable expression in smoking-related transcriptomic studies. Relative gene expression was calculated using the 2^−∆∆CT^ method [5,35,36]. Fold-change thresholds (≥2 for upregulation and ≤0.5 for downregulation) were selected based on commonly used criteria in transcriptomic screening and RT-qPCR studies [37]. Gene expression analyses were conducted exclusively between smokers adhering to and not adhering to the Mediterranean diet. No non-smoking control group was included in the experimental RT-qPCR analyses. Metabolite profiles from healthy individuals reported in the literature were incorporated at a subsequent analytical stage solely to support pathway-level interpretation and were not used for experimental or statistical comparison.

### 2.3. In Silico Analysis

Genes exhibiting significant expression changes were identified and subjected to gene enrichment analysis using the Reactome 2024 database within the EnrichR platform (https://maayanlab.cloud/Enrichr/, accessed on 12 January 2026), in order to determine associated pathways and cellular processes [38,39,40].

Metabolite data previously reported in Mediterranean diet-adherent individuals were incorporated into the analysis, referencing Papandreou et al. [41]. Differentially expressed genes were integrated with these metabolites using the Joint Pathway Analysis tool in MetaboAnalyst 6.0, and a gene–metabolite interaction map was generated to identify shared metabolic pathways affected by both gene expression changes and metabolite profiles.

Metabolite data were not experimentally measured in the present cohort. Instead, metabolite profiles derived from a previously published study involving healthy individuals adhering to the Mediterranean diet were incorporated solely for pathway-level integration and biological interpretation of the transcriptomic findings. These data were not used for direct statistical comparisons with the smoker groups.

### 2.4. Statistical Analysis

SPSS 15.0 for Windows program was used for statistical analysis. Descriptive statistics, numbers, and percentages were given for categorical variables, and mean, standard deviation, minimum, maximum, and median for numerical variables. Data are presented as mean ± standard deviation. Body weight is reported in kilograms (kg), height in centimeters (cm), and smoking duration in years. The rates in the groups were compared with the Chi-Square Test. Comparisons of numerical variables in two independent groups were made using the Student *t*-Test when the normal distribution condition was met and the Mann–Whitney U test when the normal distribution condition was not met. Spearman Correlation Analysis examined the relationship of numerical variables since the parametric test condition was not met. The statistical alpha significance level was accepted as *p* < 0.05. All graphical representations were created using GraphPad Prism (version 8.0.2, GraphPad Software, San Diego, CA, USA).

## 3. Results

### 3.1. Sociodemographic Characteristics

An equal number of people (*n*:24 adhering to the Mediterranean diet, *n*:24 not adhering to the Mediterranean diet) from both groups participated in the study. The general information obtained from participants is given in Table 1. A statistically significant difference was found in the educational status of the patients who followed the Mediterranean diet (*p* = 0.014).

The weight and body mass index (BMI) averages of the participants who adhered to the Mediterranean diet were found to have a statistically significant difference compared to those who did not adhere to the diet (*p* = 0.003 *p* < 0.001). While the mean weight of the diet-compliant group was 64.1 ± 9.5 kg, the mean BMI was 22.6 ± 2.6 kg/m^2^, the mean weight of the non-compliant group was 78.2 ± 19.0 kg, and the mean BMI was 27.4 ± 5 kg/m^2^. The mean MEDAS score of the group compliant with the Mediterranean diet was 8, while the mean MEDAS score of the non-compliant group was 3. A statistically significant difference was found between the groups in terms of adherence to diet (*p* < 0.001).

The rate of consuming less than one pack of cigarettes per day in the group who were adhering to the Mediterranean diet was statistically significantly higher than those who did not adhere to the diet (*p* = 0.046). In the diet-compliant group, individuals consuming less than one pack of cigarettes per day constituted 87.5% (*n*:21) of the group, while this rate was 62.5% (*n*:15) in the non-compliant group. According to these results, individuals who adhere to the Mediterranean diet have lower cigarette consumption on a pack-a-day basis. Mediterranean Diet Adherence Screener score level was found to be positively correlated with the education level of the participants and negatively correlated with the BMI; these findings were statistically significant (*p* < 0.001 *p* = 0.002).

As shown in Table 2, the MEDAS score level was found to be positively correlated with the education level of the participants, and positively correlated with the BMI level, at a good level and statistically significant (*p* < 0.001 *p* = 0.002).

### 3.2. Gene Expression and Enrichment Analysis

**Figure 1:**
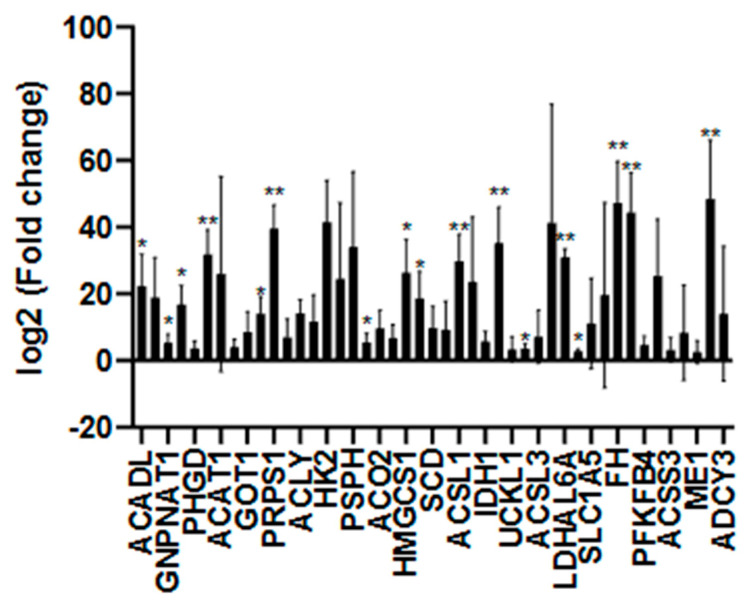
RT-qPCR analysis of genes related to energy metabolism in individuals who smoke but do not adhere to the Mediterranean diet (*n* = 24). Gene expression changes were evaluated using one-way ANOVA and compared to the control value (set as 1). Statistical significance was defined as *p* < 0.05. *p* < 0.05 (*), and *p* < 0.01 (**) versus control.

**Figure 2:**
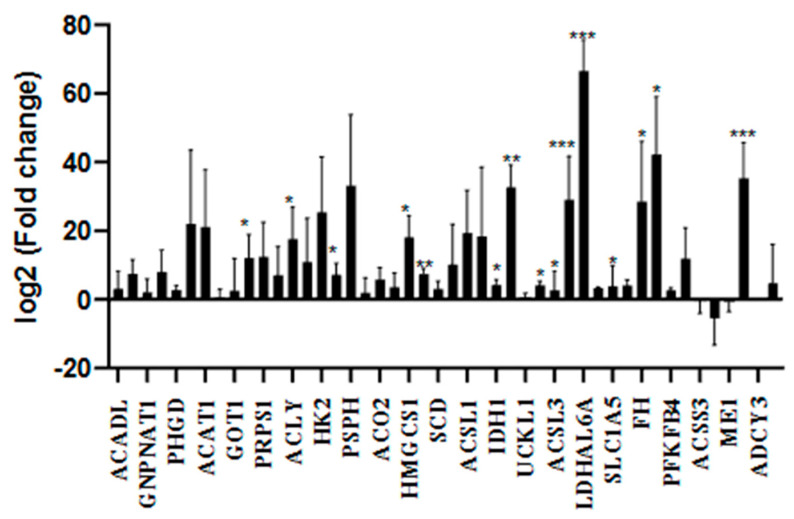
RT-qPCR analysis of genes related to energy metabolism in individuals who smoke and adhere to the Mediterranean diet (*n* = 24). Gene expression changes were evaluated using one-way ANOVA and compared to the control value (set as 1). Statistical significance was defined as *p* < 0.05. *p* < 0.05 (*), *p* < 0.01 (**), and *p* < 0.001 (***) versus control.

The impact of the Mediterranean diet on energy metabolism in smokers was evaluated using RT-qPCR with the gene panel listed in Table 1. Comparative analyses focused on differences between smokers adhering to and not adhering to the Mediterranean diet, while external reference data were used solely to support pathway-level interpretation. As shown in Figure 1 and Figure 2 and Supplementary Tables S2 and S3, both smoker groups exhibited increased expression of energy metabolism-related genes, with consistently lower expression levels observed in smokers adhering to the Mediterranean diet compared to non-adherent smokers.

Genes showing significant expression changes (≥2-fold increase or ≤0.5-fold decrease) were identified and analyzed using the Reactome 2024 database. The top 10 enriched pathways were selected based on *p*-values. As seen in Table 3 and Table 4, while both groups showed effects on carbohydrate and lipid metabolism, the Citric Acid Cycle (TCA cycle) was additionally observed in individuals adhering to the Mediterranean diet.

In conclusion, distinct differences in metabolic pathway activity were observed between the two groups. While participants not following the Mediterranean diet showed significant alterations primarily in lipid metabolism, those adhering to the diet also exhibited changes in pathways associated with the citric acid (TCA) cycle, suggesting a broader impact on energy metabolism.

### 3.3. Gene-Metabolite Interactions

**Figure 3:**
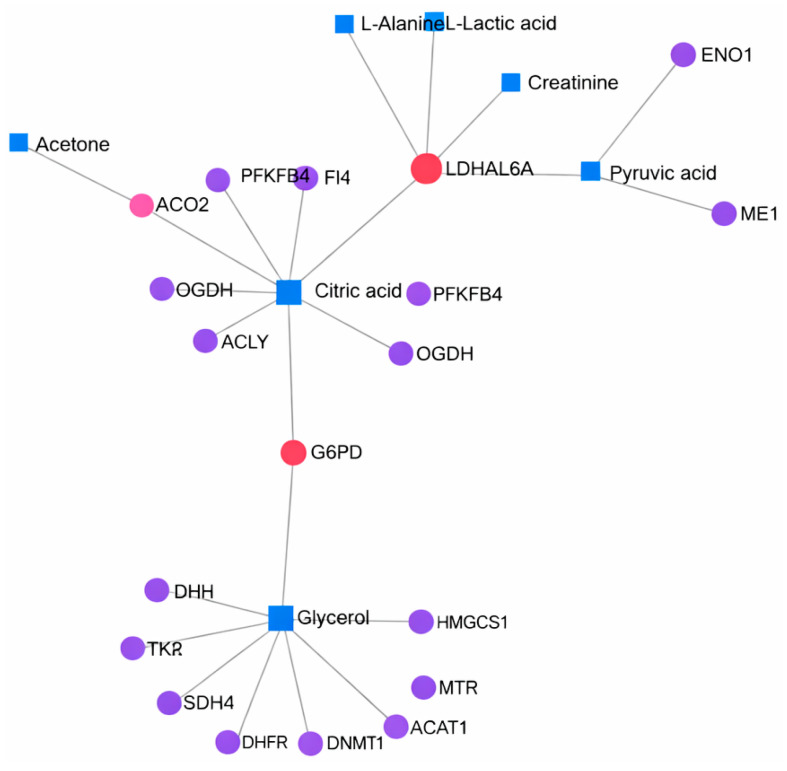
Gene–metabolite interactions in individuals not following the Mediterranean diet, analyzed using the MetaboAnalyst 6.0.2 database (accessed 22 April 2025). Nodes represent genes and metabolites involved in energy metabolism-related pathways. Node size reflects the degree of connectivity within the network, while node color indicates the type of biological entity (genes or metabolites). Edges represent pathway-level associations between genes and metabolites. This network is presented for contextual interpretation and does not represent direct experimental comparisons.

**Figure 4:**
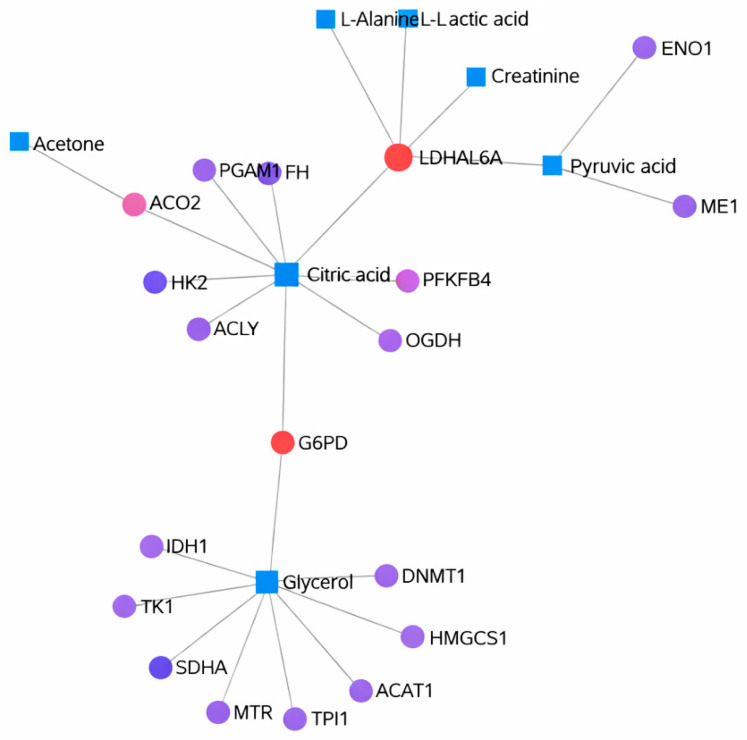
Gene–metabolite interactions in individuals following the Mediterranean diet, analyzed using the MetaboAnalyst 6.0.2 database (accessed 22 April 2025). Nodes represent genes and metabolites associated with energy metabolism pathways. Node size reflects network connectivity, node color distinguishes genes and metabolites, and edges indicate pathway-level gene–metabolite associations. The network is provided for contextual interpretation only and should not be interpreted as direct experimental comparisons.

Metabolites previously identified by Papandreou et al. in individuals adhering to the Mediterranean diet were used for the analysis, and gene–metabolite interactions were determined using the MetaboAnalyst 6.0 platform [41]. Although similar results were observed between individuals not following (Figure 3) and those adhering to the Mediterranean diet (Figure 4), notable differences were detected in certain gene–metabolite associations. In both groups, strong associations were identified between genes such as *LDHAL6A*, *G6PD*, *NME2*, *ACADL*, *OGDH*, and *GOT1*, and metabolites including L-alanine, L-lactic acid, creatinine, pyruvic acid, acetone, citric acid, and glycerol. The displayed pathway associations are intended for contextual interpretation only and should not be interpreted as direct experimental comparisons.

In conclusion, distinct differences in metabolic pathway activity were observed between the two groups. While participants not following the Mediterranean diet showed significant alterations primarily in lipid metabolism, those adhering to the diet also exhibited changes in pathways associated with the citric acid (TCA) cycle, suggesting a broader impact on energy metabolism (Table 5).

## 4. Discussion

**Figure 5:**
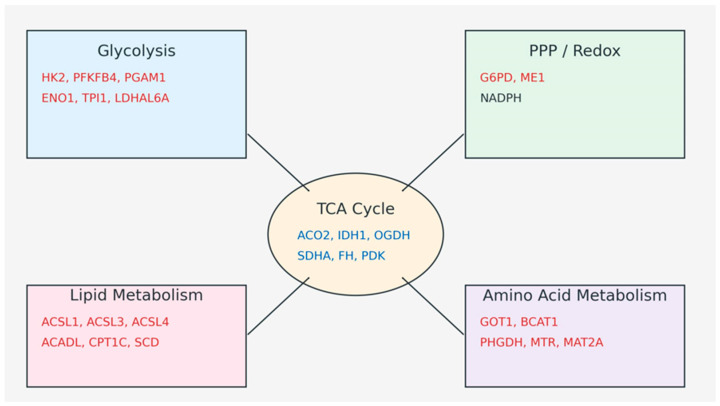
Integrated Energy Metabolism Pathways in Smokers and Mediterranean Diet Adherence. Red: Smoking-induced upregulation. Blue: Modulation by Mediterranean Diet.

Cigarette smoking is a well-established modifiable risk factor that disrupts systemic metabolic homeostasis through oxidative stress, mitochondrial dysfunction, and inflammatory signaling. In the present study, integration of transcriptomic data with metabolite associations provides a systems-level view of how adherence to the Mediterranean diet modulates smoking-induced alterations in energy metabolism pathways. The present study’s findings indicate that while smoking broadly induces upregulation across multiple metabolic pathways, adherence to the Mediterranean diet exerts pathway-specific normalizing effects rather than a uniform suppression of metabolic activity (Figure 5).

Non-communicable diseases continue to pose a significant global health burden, with smoking being a major modifiable risk factor contributing to their development. The detrimental effects of smoking, mediated largely through oxidative stress and molecular damage, underscore the urgent need for effective preventive strategies. Nutritional interventions, particularly adherence to the Mediterranean diet, have emerged as promising approaches. The Mediterranean diet is rich in fruits, vegetables, grains, seeds, olive oil, fish, and seafood and is anti-inflammatory [42], antitumorigenic [43,44], antioxidant, antiangiogenic, antiadipogenic [45,46,47], and neuroprotective [48]. It is a diet known for its protective effects against many diseases, such as the induction of apoptosis [45,49]. The present study provides molecular insights into how adherence to the Mediterranean diet may be associated with the modulation of smoking-related metabolic pathways. Specifically, differences in the expression of certain genes were observed between smokers adhering to the Mediterranean diet and those not adhering to the diet.

Assessment of socio-demographic factors and their association with MEDAS scores provides insight into dietary behavior patterns within the study population. Women were found to have higher MEDAS scores than men, reflecting a greater tendency towards healthy eating, consistent with existing literature [50]. Married individuals were found to have higher MEDAS scores than unmarried individuals across groups and genders, consistent with findings reported by Yannakoulia et al. [51]. Furthermore, an increase in education level was positively correlated with better compliance with the Mediterranean diet, likely due to improved socioeconomic status and easier access to accurate information on healthy dietary habits [52]. In agreement with the literature, the mean body weights and BMIs of individuals who adhered to the diet were found to be lower [53]. It should be noted that smokers adhering to the Mediterranean diet differed from non-adherent smokers in terms of BMI and daily cigarette consumption. Both BMI and smoking intensity are known to independently influence metabolic regulation and gene expression profiles and therefore represent potential confounding factors. Due to the cross-sectional design of the study, it was not possible to fully disentangle the effects of these variables.

It was determined that the participants who were adhering to the Mediterranean diet slept 7 h or more. Studies have shown that individuals with short-term sleep habits have a higher rate of consuming unhealthy snacks, especially foods high in fat [54]. It was observed that participants who smoked less than one pack a day had higher MEDAS scores. In addition, a study conducted by Lee et al. showed that consumption of fruit and vegetables was less in the group of adolescents who smoked compared to adolescents who did not smoke [55].

Glycolysis, one of the cellular energy pathways, is the process by which glucose taken up by cells is broken down under both anaerobic and aerobic conditions to produce ATP and certain by-products. Smokers exhibited increased expression of key glycolytic genes, including *HK2*, *PFKFB4*, *PGAM1*, *ENO1*, and *LDHAL6A*, accompanied by elevated glycolysis-associated metabolites such as pyruvate and lactate. The expression of these genes was found to be upregulated in both groups; however, a reduction was observed in the group adhering to the Mediterranean diet compared to those who did not follow the diet. Metabolite-gene interaction mapping identified glycolysis-related metabolites such as L-alanine, pyruvic acid, citric acid and glycerol. Previous studies have demonstrated that smoking increases glycolysis, thereby enhancing cellular energy production, which contributes to cellular aging [26,56,57]. Conversely, it has also been shown that smoking, depending on tissue specificity and acute or chronic exposure, can suppress glycolysis [58]. In line with these findings, the results indicate that increased expression of *SLC2A1* (glucose uptake) and *SLC2A2* (glucose transport) genes in smokers supports enhanced glucose flux. The observed suppression of glycolysis has been suggested to result from an increased ATP/ADP ratio leading to decreased energy demand and subsequent glycolytic slowdown [59]. Hexokinase 2 (HK2) is known to preserve mitochondrial outer membrane integrity while enhancing glucose metabolism to support energy production and, under conditions of nutritional deficiency, to promote cellular homeostasis and survival [60,61]. Glucose-6-phosphate dehydrogenase (G6PD), a rate-limiting enzyme in the pentose phosphate pathway, is essential for the production of NADPH and ribose-5-phosphate. Previous studies have reported an increased G6PD expression in renal cells exposed to cigarette smoke [28]. Although G6PD expression was elevated in smokers in the present study, it was reduced in individuals adhering to the Mediterranean diet, which is rich in antioxidants and may mitigate smoking-induced oxidative stress, thereby decreasing the cellular demand for G6PD. In addition to the antioxidant properties of some phytochemicals found in olive oil, they directly down-regulate the unnecessary stimulation of PI3K and MAPK-related pathways [62]. Furthermore, these phytochemicals can also activate AMP-activated protein kinase (AMPK). AMPK is an enzymatic complex that can reduce the de novo synthesis of lipids and proteins, modulate NF-κβ activation, slow down the cell cycle, and induce the production of DNA-repairing and antioxidant enzymes [63]. Overall, our findings indicate that chronic exposure to cigarette smoke increases glycolysis in smokers, whereas adherence to the Mediterranean diet attenuates this upregulation. These findings align with previous reports demonstrating that Mediterranean diet adherence improves glucose homeostasis and reduces hyperglycolytic states.

The pyruvate obtained through glycolysis is transported into the mitochondria, where it is converted into acetyl-CoA, subsequently entering the tricarboxylic acid (TCA) cycle to contribute to energy production and the synthesis of intermediate metabolites. Notably, the findings highlight a distinct pattern for the TCA cycle. Genes such as *ACO2*, *IDH1*, *OGDH*, *SDHA*, and *FH* were upregulated in smokers, but this activation was further enhanced or normalized in individuals adhering to the Mediterranean diet. Although the expression levels of these genes were increased in individuals who smoked, they were found to be even higher in those adhering to the Mediterranean diet compared to those who did not follow the diet. Alongside gene expression results, metabolite-gene interaction mapping identified citric acid as a metabolite associated with the TCA cycle. Previous studies have shown that both chronic and acute exposure to smoking impairs mitochondrial respiration [57,64]. In addition, smoking-induced disruption of pyruvate metabolism has been linked to elevated urinary lactate levels, suggesting increased mitochondrial consumption of pyruvate derived from glycolysis [27]. Previous studies have also demonstrated that adherence to the Mediterranean diet improves metabolic health by regulating glycolysis/gluconeogenesis and TCA cycle metabolites [65]. Additionally, studies suggest that the effects of nicotine exposure on glycolysis persist for an extended period even after cessation [58,66]. Rather than suppressing energy metabolism, the Mediterranean diet appears to restore efficient coupling between glycolysis and mitochondrial respiration, thereby reducing lactate accumulation and enhancing oxidative ATP production.

Smoking markedly increased the expression of lipid metabolism-related genes (*ACSL1*, *ACSL3*, *ACSL4*, *ACADL*, and *CPT1C*), along with metabolites such as acyl-CoA and acetone, indicative of enhanced fatty acid activation and β-oxidation [26,27,28,29], whereas adherence to the Mediterranean diet was associated with attenuation of this overactivation, consistent with improved lipid metabolic homeostasis [17,53]. Although increased expression levels of these genes were observed in individuals who smoked, the expression increase was found to be lower in individuals adhering to the Mediterranean diet compared to those who did not follow the diet. The Mediterranean diet includes sources of omega-3 fatty acids such as fish, which provide eicosapentaenoic acid (EPA), as well as plant-based foods rich in α-linolenic acid (ALA), including canola and soybean oils and walnuts [67]. For this reason, numerous studies have shown that lipid metabolism is regulated in individuals following the Mediterranean diet. While EPA and DHA synthesis from ALA occurs in the human body, DHA synthesis is notably limited. Foods rich in omega-3, such as fish, seafood, and walnuts, can reduce inflammation by maintaining a balanced ω-6/ω-3 ratio in the body. Additionally, the high fiber content of the Mediterranean diet (approximately 14 g per 1000 kcal) contributes to the improvement of gut microbiota, helping to reduce cholesterol and insulin levels [67]. Compared to the Western diet, the Mediterranean diet reduces intestinal permeability, thereby lowering the risk of metabolic endotoxemia [68]. These effects highlight the complex relationship between lipid metabolism and systemic inflammation. The Acyl-CoA Synthetase Long-Chain (ACSL) family, which encodes enzymes responsible for metabolizing long-chain fatty acids, plays a fundamental role in enabling the utilization of fatty acids in metabolic pathways. In a murine model study, ACSL1 expression was found to be higher in animals fed a high-fat diet and not subjected to exercise [69]. ACSL3, on the other hand, may help protect cells against oxidative stress by shielding them from lipid peroxidation through monounsaturated fatty acids (MUFAs) [70,71]. Carnitine palmitoyl transferase 1C (CPT1C) may play a role in regulating feeding behavior and whole-body energy homeostasis. An experimental study conducted in murine models with CPT1C suppression has reported reduced food intake and decreased body weight; however, these models also exhibited a pronounced susceptibility to weight gain when exposed to a high-fat diet [72]. This suggests that CPT1C facilitates the storage of acyl-CoAs in triacylglycerols rather than promoting their mitochondrial oxidation [64,73]. A study showed that propionate is activated by acyl-CoA synthetase short-chain family member 3 (ACSS3), located on the mitochondrial inner membrane in brown adipocytes. Silencing the ACSS3 gene led to decreased brown adipose tissue, increased white adipose tissue, and consequently, glucose intolerance and insulin resistance. This condition became even more pronounced when combined with a high-fat diet [69,74]. Stearoyl-CoA desaturase (SCD) regulates the expression of proteins involved in lipogenesis and mitochondrial fatty acid oxidation and also contributes to the synthesis of membrane phospholipids, cholesterol esters, and triglycerides [75,76]. In the present study, the relatively suppressed expression of genes involved in lipid metabolism in individuals adhering to the Mediterranean diet suggests that the Mediterranean diet may help maintain lipid homeostasis.

Amino acid metabolism plays a central role in numerous cellular processes, including the synthesis of protein building blocks required for cellular function maintenance, energy production, neurotransmitter synthesis, hormone synthesis, and immune responses. Alterations in amino acid metabolism were evident in smokers, with increased expression of genes involved in transamination, one-carbon metabolism, and amino acid biosynthesis (*GOT1*, *BCAT1*, *PHGDH*, *MTR*, and *MAT2A*). These changes were accompanied by elevated levels of alanine and glutamate, reflecting increased amino acid turnover to support energy production and redox balance under smoking-induced stress. Elevated expression of these genes was observed in smokers regardless of diet, whereas a decrease in expression was noted in individuals adhering to the Mediterranean diet. Previous studies have shown that smoking disrupts amino acid metabolism, increasing the risk of insulin resistance and cardiovascular diseases [29,77,78,79]. Thus, the observed alterations in amino acid metabolism among smokers in present study are consistent with existing literature. Conversely, a study investigating patients on the Mediterranean diet reported reductions in plasma metabolites such as glucose and insulin, along with modulatory effects on amino acids [78]. While aromatic and branched-chain amino acids are associated with various diseases, the Mediterranean diet enriched with extra virgin olive oil has been shown to reduce elevated branched-chain amino acid levels in type 2 diabetes patients, thereby contributing to metabolic regulation [78,79].

Collectively, the summarized data indicate that smoking induces a coordinated upregulation of glycolysis, lipid metabolism, amino acid turnover, and redox pathways. The present findings do not establish a causal relationship; rather, they indicate associations between dietary adherence and metabolic gene expression patterns in smokers. Adherence to the Mediterranean diet does not appear to uniformly suppress these pathways but may be associated with differential regulation of energy metabolism-related processes. Enhanced TCA cycle activity, reduced glycolytic and lipid overactivation, and improved redox homeostasis suggest that the Mediterranean diet promotes metabolic resilience in smokers. These findings provide molecular evidence supporting the Mediterranean diet as a viable nutritional strategy to mitigate smoking-induced metabolic dysregulation and underscore the importance of dietary patterns in modulating gene–metabolite networks associated with chronic disease risk. The integration of transcriptomic data with literature-derived metabolite profiles was intended to provide biological context rather than to imply equivalence between smokers and healthy individuals. The observed gene–metabolite patterns indicate coordinated modulation of energy metabolism pathways and may reflect differences in metabolic efficiency and redox demand. These findings support the biological plausibility of the results without implying direct metabolic normalization.

Several limitations of this study should be acknowledged. First, differences in BMI and cigarette consumption between smokers adhering to and not adhering to the Mediterranean diet may have contributed to the observed gene expression patterns independently of dietary effects. Due to the limited sample size, formal multivariable adjustment was not performed. Therefore, the findings should be interpreted as associative rather than causal.

## 5. Conclusions

In conclusion, the pathway-level analysis indicates that cigarette smoking is associated with coordinated activation of genes involved in glycolysis, lipid and amino acid metabolism, the pentose phosphate pathway, and redox regulation, reflecting increased metabolic and oxidative stress. Adherence to the Mediterranean diet was associated with selective attenuation of these smoking-related alterations, characterized by moderated activation of catabolic pathways, improved engagement of the tricarboxylic acid cycle, and reduced oxidative stress burden. Overall, these findings suggest that the Mediterranean diet enhances metabolic resilience in smokers by rebalancing key gene–metabolite networks involved in energy metabolism and redox homeostasis, highlighting its potential relevance in the context of smoking-related cardiometabolic risk.

## Figures and Tables

**Table 1:** General Information of Participants. ^≠^ Mediterranean Diet Compliance Scores; ^¥^ Chi-Square Test; * Student *t* Test ^#^ Mann–Whitney U Test.

		Groups		*p*
		Adhering to the Mediterranean Diet	Not Adhering to the Mediterranean Diet	
		Ave. ± SD	Ave. ± SD	
		Min–Max. (Median)	Min–Max. (Median)	
**Age**		34.3 ± 8.1	32.8 ± 9.7	0.565 *
		24–49 (32.5)	18–52 (32.5)	
**Gender** *n* (%)	Female	19 (79.2)	14 (58.3)	0.119 ^¥^
	Male	5 (20.8)	10 (41.7)	
**Marital Status** *n* (%)	Married	15 (62.5)	13 (54.2)	0.558 ^¥^
	Single	9 (37.5)	11 (45.8)	
**Educational Status** *n* (%)	Primary School	0 (0.0)	1 (4.2)	0.014 ^¥^
	Middle School	-	-	
	High School	3 (12.5)	11 (45.8)	
	University	15 (62.5)	11 (45.8)	
	Graduate School	6 (25.0)	1 (4.2)	
**Weight**		64.1 ± 9.5	78.2 ± 19.0	0.003 *
		46–83 (66)	46–120 (84)	
**Height**		168.1 ± 7.8	168.1 ± 12.6	0.989 *
		158–184 (168)	150–200 (166)	
**BMI**		22.6 ± 2.6	27.4 ± 5.2	<0.001 *
		18.3–27.3 (22.8)	17.3–39.5 (28.7)	
**Cigarette (Package)** *n* (%)	<1 package/day	21 (87.5)	15 (62.5)	0.046 ^¥^
	≥1 package/day	3 (12.5)	9 (37.5)	
**Age to Start Smoking**		19.1 ± 3.6	20.3 ± 5.1	0.501 ^#^
		14–28 (18)	13–31 (20)	
**Smoking Duration**		3.50 ± 0.72	3.21 ± 1.10	0.503 ^#^
		1–5 years and >10 years (>10 years)	<1 year and >10 years (>10 years)	
**Exercise** *n* (%)	Exercising	9 (37.5)	10 (41.7)	0.768 ^¥^
	Not exercising	15 (62.5)	14 (58.3)	
**Sleep** *n* (%)	<7 h/day	13 (54.2)	16 (66.7)	0.376 ^¥^
	≥7 h/day	11 (45.8)	8 (33.3)	
**MEDAS scores** ^≠^		8.17 ± 1.13	3.54 ± 1.22	<0.001 ^#^
		7–10 (8)	1–6 (3)	

**Table 2:** The Relationship Between Some of the Participants’ Characteristics and MEDAS Scores. * Spearman Correlation Analysis.

	MEDAS	
	r	*p* *
**Age**	0.143	0.333
**Education**	0.560	<0.001
**BMI**	−0.445	0.002
**Age to Start Smoking**	0.023	0.878
**Smoking Duration**	0.181	0.219

**Table 3:** Gene enrichment analysis results for individuals not following the Mediterranean diet were obtained using the Reactome 2024 database. The top 10 associated pathways are listed based on statistical significance (*p*-values) (Accessed date 22 April 2025).

Index	Name	*p*-Value	Adjusted *p*-Value	Odds Ratio	Combined Score
**1**	Metabolism	1.216 × 10^−20^	2.273 × 10^−18^	28.95	1327.74
**2**	Fatty acyl-CoA Biosynthesis	5.915 × 10^−9^	5.530 × 10^−7^	100.46	1903.37
**3**	Metabolism of Lipids	5.985 × 10^−7^	0.00002730	9.88	141.58
**4**	Fatty Acid Metabolism	6.671 × 10^−7^	0.00002730	23.29	331.15
**5**	Metabolism of Carbohydrates	7.299 × 10^−7^	0.00002730	16.79	237.20
**6**	Integration of Energy Metabolism	0.000001380	0.00004301	31.10	419.66
**7**	Aerobic Respiration and Respiratory Electron Transport	0.000006398	0.0001709	15.52	185.61
**8**	Glycolysis	0.000009297	0.0002173	35.52	411.59
**9**	Synthesis of Very Long-Chain Fatty acyl-CoAs	0.00001056	00002194	86.33	989.23
**10**	Glucose Metabolism	0.00001613	0.0003017	30.68	338.59

**Table 4:** Gene enrichment analysis results for individuals following the Mediterranean diet were obtained using the Reactome 2024 database. The top 10 associated pathways are listed based on statistical significance (*p*-values). (Accessed date 22 April 2025).

Index	Name	*p*-Value	Adjusted *p*-Value	Odds Ratio	Combined Score
**1**	Metabolism	8.928 × 10^−24^	1.679 × 10^−21^	27.36	1452.08
**2**	Citric Acid Cycle (TCA)	9.596 × 10^−9^	9.021 × 10^−7^	90.42	1669.28
**3**	Fatty acyl-CoA Biosynthesis	1.496 × 10^−8^	9.376 × 10^−7^	81.93	1476.17
**4**	Aerobic Respiration and Respiratory Electron Transport	7.166 × 10^−8^	0.000003368	17.87	294.03
**5**	Metabolism of Carbohydrates	1.656 × 10^−7^	0.000006228	15.95	248.99
**6**	Glycolysis	5.215 × 10^−7^	0.00001634	37.93	548.65
**7**	Glucose Metabolism	0.000001043	0.00002803	32.69	450.27
**8**	Fatty Acid Metabolism	0.000001984	0.00004663	18.87	247.83
**9**	Integration of Energy Metabolism	0.000003419	0.00006675	25.36	319.22
**10**	Metabolism of Lipids	0.000003550	0.00006675	7.78	97.65

**Table 5:** Integrated gene–metabolite interactions and affected metabolic pathways in smokers according to Mediterranean diet adherence. (Arrow displays enhancement of gene expression).

Pathway	Key Genes	Associated Metabolites	Direction in Smokers	Effect of Mediterranean Diet
Glycolysis	*HK2*, *PFKFB4*, *PGAM1*, *ENO1*, *LDHAL6A*	Pyruvate, lactate, glycerol	↑	Partial attenuation
Glucose Transport	*SLC2A1*, *SLC2A2*	Glucose	↑	Reduced expression
TCA Cycle	*ACO2*, *IDH1*, *OGDH*, *SDHA*, *FH*	Citric acid	↑	Further activation/normalization
Lipid Metabolism	*ACSL1*, *ACSL3*, *ACSL4*, *ACADL*, *CPT1C*	Acyl-CoA, acetone	↑	Suppressed overactivation
Amino Acid Metabolism	*GOT1*, *BCAT1*, *PHGDH*, *MTR*, *MAT2A*	Alanine, glutamate	↑	Reduced turnover
PPP/Redox	*G6PD*, *ME1*	NADPH-related metabolites	↑	Decreased oxidative demand
Energy Integration	*PDK*, *NME2*	ATP-related metabolites	↑	Improved coupling

## Data Availability

The original contributions presented in the study are included in the article/Supplementary Materials, further inquiries can be directed to the corresponding author.

## Supplementary Materials

The following supporting information can be downloaded at: https://www.mdpi.com/article/10.3390/nu18020276/s1, Figure S1: Consort flow chart of the study; Table S1: RT-qPCR Primer Sets Used in the Study; Table S2: RT-qPCR results of individuals who smoke but do not adhere to the Mediterranean diet; Table S3: RT-qPCR results of individuals who smoke and adhere to the Mediterranean diet.

## Abbreviations

The following abbreviations are used in this manuscript:

WHO	World Health Organisation
GBD	Global Burden of Disease
UDP-GlcNac	Uridine diphosphate *N*-acetylglucosamine
R-5P	D-Ribose 5-Phosphate
NO	Nitric Oxide
OL	Oleuropein
MEDAS	Mediterranean Diet Adherence Screener
MMA	Methyl Malonic Acid
LPO	Lipid Peroxide
