# Supplementary material for: Adherence to the Mediterranean Diet and Metabolic Gene Expression in Smokers: An Integrative Transcriptomic Approach
Source: Nutrients. 2026 Jan 15;18(2):276. doi: 10.3390/nu18020276 (PMC12845490; doi:10.3390/nu18020276)
Supplement: Supplementary file 1 [file nutrients-18-00276-s001.zip › nutrients-4078333-supplementary.pdf]

Adherence to the Mediterranean Diet and Metabolic Gene Ex-pression in Smokers An Integrative Transcriptomic Approach

Supplementary Files

Suplementary Figure S1: Consort flow chart of the study.

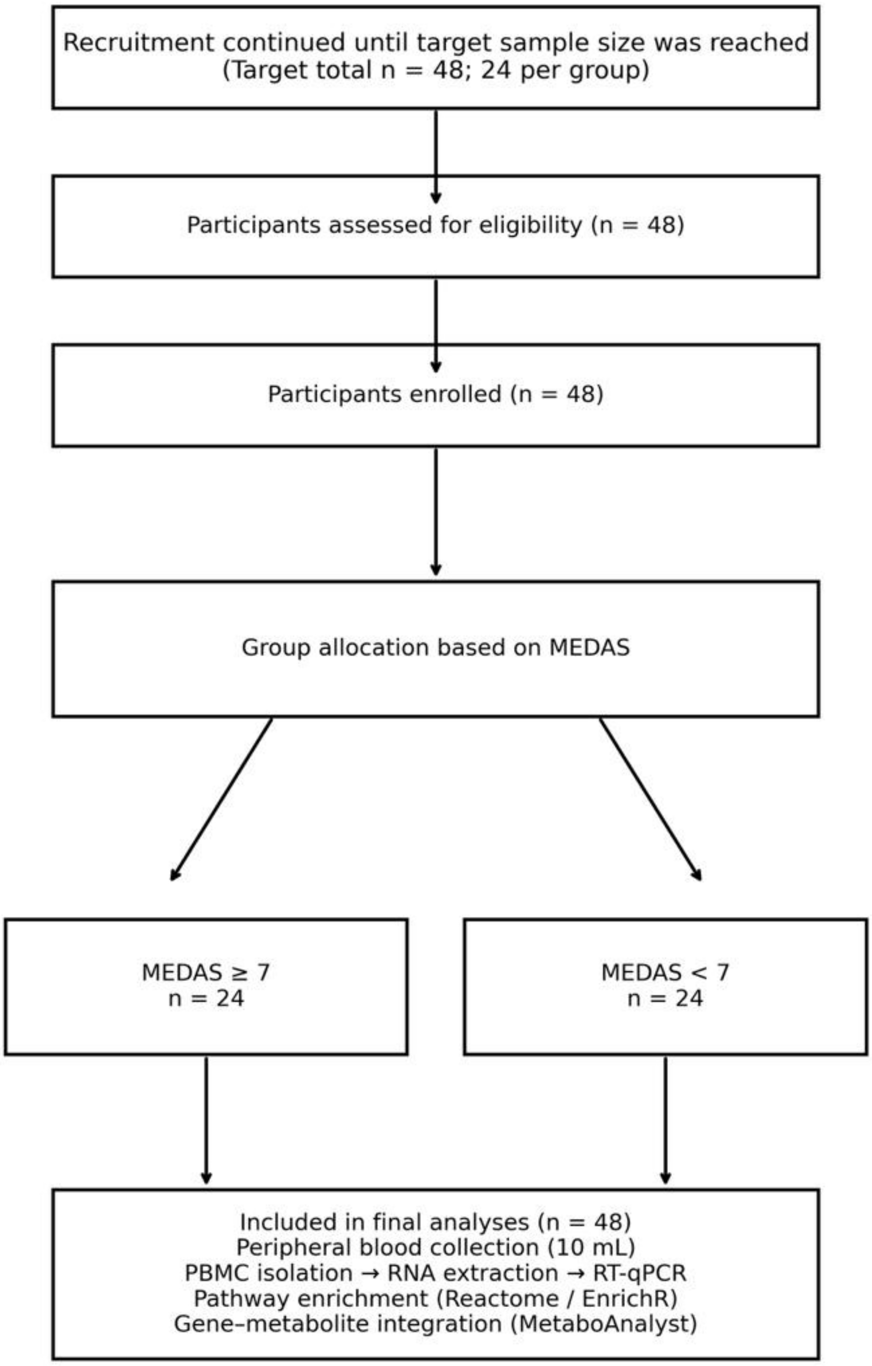

Supplementary Table S1: RT-qPCR Primer Sets Used in the Study.

**Metababolic Pathway**	**Associated Genes**
Carbohydrate Metabolism (Glycolysis and Glucose Handling)	*HK2, PFKFB4, TPI1, ENO1, PGA1, PDK, SLC2A1, SLC2A2*
Amino Acid Metabolism and Transamination	*BCAT1, GOT1, PHGD, PSPH*
Serine–Glycine / One-Carbon Metabolism	*MTR, MAT2A, DHFR*
Pentose Phosphate Pathway and NADPH Metabolism	*G6PD, TKTL1, PRPS1*
Mitochondrial Energy Metabolism (TCA Cycle and ETC Interface)	*ACO2, OGDH, IDH1, FH, SDHA*
Lipid Metabolism	*ACSL1, ACSL3, ACSL4, SCD, ACADL, CPT1C, ACLY, ACAT1, ACSS3, HMGCS1, PAFAH1B2*
Nucleotide Metabolism and Salvage Pathways	*TK1, UCKL1, NME2, AMPD3, GNPNAT1*
Solute Carrier–Mediated Amino Acid Transport	*SLC1A5, SLC7A1*
Signal Transduction Linked to Metabolic Regulation	*PDE4D, ADCY3*

Supplementary Table S2: RT-qPCR results of individuals who smoke but do not adhere to the Mediterranean diet. The table presents the mean and standard deviation (SD) values for each gene expression analyzed using the $2^{-\Delta\Delta Ct}$ Livak method (n = 24).

Gene	Expression Change	Gene	Expression Change	Gene	Expression Change
ACADL	22.34 ± 9.5	***OGDH***	24.44 ± 22.85	***ACSL3***	7.13 ± 7.87
AMPD3	18.84 ± 12.03	***PSPH***	34.02 ± 22.59	***ENO1***	41.27 ± 35.55
GNPNAT1	5.3 ± 2.57	***TKTL1***	5.42 ± 2.75	***LDHAL6A***	30.95 ± 2.48
MTR	16.88 ± 55	***ACO2***	9.63 ± 5.45	***PDK***	2.75 ± 0.7
PHGD	3.63 ± 2.27	***DHFR***	6.72 ± 4.03	***SLC1A5***	11.11 ± 13.55
SLC7A1	31.84 ± 7.35	***HMGCS1***	26.32 ± 9.962	***ACSL4***	19.62 ± 27.73
ACAT1	25.91 ± 29.23	***PAFAH1B2***	18.67 ± 8.03	***FH***	47.3 ± 12.31
BCAT1	4.00 ± 2.34	***SCD***	9.79 ± 6.51	***MAT2A***	44.33 ± 12.03
GOT1	8.52 ± 6.1	***TPI1***	9.29 ± 8.48	***PFKFB4***	4.64 ± 2.66
NME2	14.04 ± 5.14	***ACSL1***	29.72 ± 8.13	***SLC2A1***	25.34 ± 19.97
PRPS1	39.68 ± 6.82	***DNMT1***	23.61 ± 19.45	***ACSS3***	3.17 ± 3.65
TK1	6.99 ± 5.44	***IDH1***	5.7 ± 3.0	***G6PD***	8.38 ± 14.26
ACLY	14.10 ± 4.17	***PDE4D***	35.25 ± 10.7	***ME1***	2.56 ± 3.4
CPT1C	11.61 ± 8.01	***UCKL1***	3.33 ± 3.81	***PGAM1***	48.49 ± 17.54
HK2	41.44 ± 12.46	***SDHA***	3.6 ± 1.41	***ADCY3***	14.07 ± 20.18

Supplementary Table S3: RT-qPCR results of individuals who smoke and adhere to the Mediterranean diet. The table presents the mean and standard deviation (SD) values for each gene expression analyzed using the $2^{-\Delta\Delta Ct}$ Livak method (n = 24).

**Gene**	**Expression Change**	**Gene**	**Expression Change**	**Gene**	**Expression Change**
ACADL	3.1 ± 5.1	***OGDH***	7.19 ± 3.37	***ACSL3***	2.62 ± 5.6
AMPD3	7.43 ± 4.15	***PSPH***	33.14 ± 20.65	***ENO1***	29.04 ± 12.64
GNPNAT1	2.0 ± 3.96	***TKTL1***	1.99 ± 4.34	***LDHAL6A***	66.51 ± 9.1
MTR	8.0 ± 6.38	***ACO2***	5.76 ± 3.54	***PDK***	3.31 ± 0.35
PHGD	2.74 ± 1.28	***DHFR***	3.5 ± 4.21	***SLC1A5***	3.91 ± 5.96
SLC7A1	22.06 ± 21.5	***HMGCS1***	18.14 ± 6.31	***ACSL4***	4.08 ± 1.65
ACAT1	21.15 ± 16.65	***PAFAH1B2***	7.41 ± 1.64	***FH***	28.52 ± 17.54
BCAT1	0.55 ± 2.44	***SCD***	3.07 ± 2.21	***MAT2A***	42.24 ± 16.81
GOT1	2.52 ± 9.41	***TPI1***	10.2 ± 11.65	***PFKFB4***	2.6 ± 0.82
NME2	12.07 ± 6.84	***ACSL1***	19.35 ± 12.46	***SLC2A1***	11.84 ± 9.01
PRPS1	12.35 ± 10.11	***DNMT1***	18.34 ± 20.2	***ACSS3***	-0.4 ± 3.66
TK1	7.07 ± 8.28	***IDH1***	4.17 ± 1.61	***G6PD***	-5.49 ± 7.83
ACLY	17.66 ± 9.21	***PDE4D***	32.67 ± 6.5	***ME1***	-0.62 ± 2.9
CPT1C	10.85 ± 12.8	***UCKL1***	0.65 ± 1.13	***PGAM1***	35.31 ± 10.44
HK2	25.34 ± 16.23	***SDHA***	4.09 ± 1.17	***ADCY3***	4.68 ± 11.39
